# The Unique Genetic and Histological Characteristics of DMBA-Induced Mammary Tumors in an Organoid-Based Carcinogenesis Model

**DOI:** 10.3389/fgene.2021.765131

**Published:** 2021-11-29

**Authors:** Mie Naruse, Rikako Ishigamori, Toshio Imai

**Affiliations:** Central Animal Division, National Cancer Center Research Institute, Tokyo, Japan

**Keywords:** mammary tissue, organoids, carcinogenesis, DMBA, mutations, adenocarcinoma, squamous cell carcinoma, nude mouse

## Abstract

Here, we report a model system using *in vitro* 7,12-dimethylbenz[*a*]anthracene (DMBA; 0.6 μM)-treated mammary tissue-derived organoids generated from heterozygous BALB/c-*Trp53* knockout mice to induce tumors after injection into the nude mouse subcutis. In parallel, a single oral dose of DMBA (50 mg/kg bodyweight) to the same murine strain induced mammary adenocarcinomas, characterized by biphasic structures differentiated into luminal and myoepithelial lineages and frequent *Hras* mutations at codon 61. In the present study, the genetic and histological characteristics of DMBA-induced tumors in the organoid-based model were evaluated to validate its similarities to the *in vivo* study. The organoid-derived tumors were low-grade adenocarcinomas composed of luminal and basal/myoepithelial cells. When the organoid-derived carcinomas were passaged to other nude mice, they partly progressed to squamous cell carcinomas (SCCs). Whole exome sequencing revealed no mutations at *Hras* codon 61 in the organoid-derived tumors. However, various mutations were detected in other genes such as *Tusc3* and *Tgfbr2*, which have been reported as cancer-associated or homeostatic squamous cell genes. The most common mutational pattern observed in these genes were the G:C to T:A transversions and G:C to A:T transitions, which are not typical of the mutations caused by DMBA treatment. In conclusion, DMBA exhibited carcinogenicity in the both the *ex vivo* and *in vivo* mammary carcinogenesis models, albeit with distinct histological and genetical alterations. Further studies are needed to clarify whether organoid-based carcinogenesis models generated following chemical treatment *in vitro* could be applied to the clarification of the novel mode of action of chemical carcinogenesis.

## Introduction

A method for the long-term expansion of three dimensional (3D) organoids derived from healthy intestinal tissue was first introduced by Sato and Clevers et al. (Sato et al., 2009). Subsequently, various healthy and tumor tissue-derived organoids, including those from mammary tissues/carcinomas, have been established (Jarde et al., 2016; Sumbal et al., 2020). It is now possible to generate organoids that share many architectural features with their tissue of origin, and contain not only stem cells but also various types of differentiated cells such as enterocytes, goblet cells, Paneth cells, and enteroendocrine cells as is the case for intestinal organoids (Sato et al., 2009), or myoepithelial and luminal cells (including β-casein expressing cells) as applies to mammary organoids (Sumbal et al., 2020). As great preponderance target organs, liver, kidney, lung, and thyroids have been demonstrated in chemical toxicology study using *in vivo* systems (Martin et al., 2009). 3D-cultured organoid models of these organs have been generated. For example, liver organoids with bile transport function were developed from pluripotent stem cell lines, in which the hepatocyte transcriptomic state relative to primary hepatocytes was confirmed (Shinozawa et al., 2021). These *in vitro* organoid systems should be more readily applied to the development of mechanism-based toxicological studies. To this end, we recently reported an organoid-based chemical carcinogenesis model employing organoids derived from healthy murine tissue (Naruse et al., 2020). In that study, four genotoxic chemicals, including 7,12-dimethylbenz[a]anthracene (DMBA), were administered *in vitro* to lung, liver (biliary tract), and/or mammary organoids *Trp53* to examine their tumorigenicity after injection into nude mice, and organoids with a heterozygous *Trp53* knockout background partly showed higher susceptibilities than those with wild type background. The four chemicals exhibited tumorigenicity or carcinogenic histopathological characteristics accompanied by the activation of oncogenic kinases, echoing previous reports from corresponding animal studies.

As one of the most potent carcinogenic polycyclic aromatic hydrocarbons, DMBA is renowned for its properties as a mammary carcinogen that induces mammary adenocarcinomas in both rats and mice (Papaconstantinou et al., 2006; Imai et al., 2013; Abba et al., 2016). The molecular characteristics of DMBA-induced mammary carcinogenesis have been widely evaluated. In mammary carcinomas induced in female Sprague-Dawley rats by a single oral dose of DMBA, hormone receptors (HRs), such as the estrogen receptor α (ERα) and/or the progesterone receptor, exhibited positive (Alvarado et al., 2017) and frequent *Hras* mutations in codon 61 (El-Sohemy and Archer, 2000). On the other hand, in CD2F1 (BALB/c × DBA/2) female mice, different DMBA-treatment latencies resulted in controversial genetic patters (Abba et al., 2016); a short-latency treatment predominantly induced *Hras* mutations, while a long-latency treatment induced *Pten* and *Pik3ca* mutations. In addition, carcinomas arising from the long-latency treatment were predominantly HRs-positive, while carcinomas induced by the short-latency treatment were HRs-positive or -negative in equal measure. We previously reported that a single oral dose of DMBA administered to heterozygous BALB/c *Trp53* knockout female mice significantly accelerated induction of ERα-positive mammary carcinomas with frequent *Hras* mutations at codon 61 (10/10 mice) as compared with BALB/c wild type mice, suggesting the combination of *Trp53* gene function deficiency and exogenous factors contributed the increased susceptibility to mammary carcinogenesis (Machida and Imai, 2021). In addition, mammary carcinomas induced by a single DMBA dose administered to heterozygous BALB/c *Trp53* knockout mice were characterized by biphasic structures with luminal and myoepithelial cells (Machida and Imai, 2021). This former system, with its distinct genetic and histological properties, was considered to be appropriate for a validation study of an organoid-based chemical carcinogenesis model comparing their similarities.

In the present study, histological and genetic mutational characteristics of tumor tissues, induced by DMBA in the organoid-based carcinogenesis model, were evaluated to validate its similarities to the aforementioned *in vivo* study. We found that DMBA exhibited carcinogenicity in the both *ex vivo* and *in vivo* mammary carcinogenesis models, albeit with distinct histological and genetical alterations. However, the properties of the two model systems differed, possibly because DMBA-induced gene mutations underwent selective pressure in our chosen organoid culture conditions and the mutated cells expanded clonally in the mouse subcutis.

## Materials and Methods

An outline of samples used in the present study is provided in Supplementary Figures S1A,B.

### Organoids

In the present study, we used organoids that were already established and cryopreserved (Supplementary Figure S1A), prior to treatment with 7,12-dimethylbenz[*a*]anthracene (DMBA; Sigma-Aldrich, St. Louis, MO, United States) as previously described (Naruse et al., 2020). In brief, the organoids were generated from normal mammary tissue derived from heterogeneous BALB/c-*Trp53* knockout female mice (Machida and Imai, 2021) at 5 weeks of age, and cultured in advanced DMEM/F12 medium (Thermo Fisher Scientific, Waltham, MA, United States) supplemented with penicillin-streptomycin (Wako Pure Chemical, Osaka, Japan), amphotericin B (Wako), l-glutamine (Wako), 50 ng/ml murine EGF (Peprotech, Rocky Hill, NJ, United States), and 0.2–1.0% bovine serum albumin (Wako). Additional reagents for initial organoid establishment included 100 ng/ml Noggin (Peprotech), 250 ng/ml R-Spondin (R&D Systems, Minneapolis, MN, United States), 10 μM Y27632 (Wako), and 1 μM Jagged-1 (AnaSpec, Fremont, CA, United States). The organoids were subsequently maintained in medium containing 1 µM Y27632, 0.125 µM A83-01 (Focus Biomolecules, Plymouth Meeting, PA, United States), and 40 ng/ml FGF10 (GenScript, Piscataway, NJ, United States). During the process of organoid establishment and passage, cells were seeded on a Matrigel (Corning, Bedford, MA, United States) and incubated overnight at 37°C. One day later, viable cells attached to the Matrigel were sandwiched in an additional Matrigel layer and submerged in media to resume 3D culture (MBOC; Matrigel Bilayer Organic Culture) (Maru et al., 2019). During each passage, organoids were collected using a cell scraper, washed with PBS, and treated with Accumax (Innovative Cell Technologies, San Diego, CA, United States) to create a single cell suspension. Passaging was conducted every 5–10 days at a dilution of 1:3.

### Chemical Treatment of Organoids

DMBA treatment of mammary organoids was conducted at concentrations of 0 (in a 0.5% dimethyl sulfoxide vehicle; Wako), 0.2, and 0.6 µM for 24 h, which was repeated three times after passaging of the organoids. During the process of DMBA treatment, S9 mix (50 μg/ml of S9 protein: S-9/Cofactor C set; Oriental Yeast, Tokyo, Japan) was added to the medium, for metabolic activation of DMBA to their carcinogenic properties (Gocke et al., 2009). Organoids that were either left untreated or treated with 0.6 µM DMBA (since tumorigenicity of the DMBA-treated organoids was observed at a DMBA concentration of 0.6 µM but not 0.2 µM) were used in further experiments (Naruse et al., 2020). Organoids cryopreserved after treatment with the Cell Recovery Solution (Corning) according to the manufacturer’s instructions were used for whole exome sequencing analysis (WES), while those cryopreserved in LaboBanker 2 medium (TOSC Japan, Tokyo, Japan) and stored at −80°C were injected into nude mice in the tumorigenicity assay.

### Organoid-Derived Tumor Tissues

Organoid-derived tumor tissues induced by DMBA treatment after subcutaneous injection into BALB/c-*nu*/*nu* female mice (5 weeks of age; CLEA Japan, Inc., Tokyo, Japan) were previously harvested and cryopreserved (Supplementary Figure S1A) (Naruse et al., 2020). In the present study, the tumor tissue samples were used for whole genome sequencing (WES).

### Whole Exome Sequencing (WES), *Hras* Codon 61 Targeting Digital PCR, and Sanger Sequencing Analyses

Genomic DNA extracted from *in vitro* 0.6 μM DMBA-treated organoids or DMBA-treated organoid-derived subcutaneous tumor tissues injected into the subcutis of nude mice (diagnosed as adenocarcinoma), were prepared using the NucleoSpin Tissue kit (Takara Bio, Kusatsu, Japan) according to the manufacturer’s protocol. Fragment libraries were created from the genomic DNA sheared into 150–200 bp fragments using the Covaris S220 ultrasonicator (Covaris Biotechnology. Woburn, MA, United States). Target enrichment was then performed according to the manufacturer’s protocol (Agilent SureSelect Mouse All Exome kit; Agilent Technologies, Santa Clara, CA, United States). Captured DNA was amplified followed by solid-phase bridge amplification and paired-end sequenced on the Illumina Hiseq 2500 (Illumina, San Diego, CA, United States). Alignment of reads to the mouse reference sequence (mm10 assembly) and variant detection was performed using the Genome Analysis Toolkit 3.4 (GATK, www.broadinstitute.org/gark). Approximately 125–175 million PE reads were mapped to the mouse reference sequence. Mean target depth was >200. Visual inspection of read alignments for confirmation and validation of the WES data were performed using the Integrative Genomics Viewer (IGV) (Robinson et al., 2017). Absolute quantification of mutations at *Hras* codon 61 was performed using the QuantStudio 3D Digital PCR System together with the Gene Amp PCR System 9700 (Thermo Fisher Scientific, Waltham, MA United States) according to the manufacturer’s instructions. Briefly, wild type *Hras* alleles (*Hras* codon 61: CAA) were represented by a VIC probe while mutant *Hras* alleles (*Hras* codon 61: CTA) were represented by a FAM probe. ID numbers of primers for the Custom TaqMan SNP Genotyping Assay were ANNKZ63 (Thermo Fisher Scientific). The 14.5 μl PCR reaction mixture containing 1× QuantStudio 3D Digital PCR Master Mix v2, 1× TaqMan Assay, and 50 ng DNA was loaded onto the QuantStudio 3D Digital PCR 20K Chips v2 using the QuantStudio 3D Digital PCR Chip Loader. The loaded chips underwent amplification using the Gene Amp PCR System 9700 under the following conditions: 96°C for 10 min, followed by 39 cycles of 60°C for 2 min and 98°C for 30 s, 60°C for 2 min and then 4°C. After PCR reaction completion, the chips were imaged on the QuantStudio 3D digital PCR instrument and analyzed with the aid of QuantStudio 3D AnalysisSuite Cloud Software v3.1 (Thermo Fisher Scientific).

To confirm the presence of a mutation at *Vps13d* codon 295 (which was detected by WES in the both DMBA-treated organoids and the organoid-derived tumor tissues induced by *in vitro* DMBA treatment) and another mutation at *Tgfbr2* codon 549 (which was detected by WES in the organoid-originated tumor tissues induced by *in vitro* DMBA treatment but not in the DMBA-treated organoids), Sanger sequencing analyses were performed using DNA isolated by the same method from tumor tissues passaged into the nude mouse subcutis. Meanwhile, to confirm the presence or absence of mutations at *Hras* codon 61 (which were observed in DMBA-induced mammary carcinoma tissues in a previous *in vivo* study), we also performed Sanger sequencing analyses of DNA samples (isolated using the same method) from organoid-derived tumor tissues passaged into the nude mouse subcutis. The PCR products, including mutations, were amplified using specific PCR primers, and the amplified PCR products were directly sequenced using the forward primers used in the PCR reaction for each target. Primers used for *Vps13d* codon 295, *Tgfbr2* codon 549, and *Hras* codon 61 were forward, 5′ CTG​ATG​CTT​TGG​TCC​TGG​AGT 3’; reverse, 5′ AAA​GGA​CAG​GGA​GAG​CAT​GC 3′, forward, 5′ GGT​AGT​GTT​CAG​CGA​GCC​AT 3’; reverse, 5′ CAT​GGA​GAC​CAC​CCA​CTG​AC 3′ and forward, 5′ AAA​CAG​GTA​GTC​ATT​GAT​GG 3’; reverse, 5′ GCA​AAT​ACA​CAG​AGG​AAG​CC 3′, respectively.

For a copy number alteration (CNA) analysis, reads counts which mapped to mouse genome (mm10) were calculated and normalized using Cufflinks and Cuffnorm in the Galaxy Platform (https://galaxyproject.org/) with default setting, and a frequency plot was generated based on the log2 ratios of 0.6 μM DMBA-treated organoids to DMBA-untreated control organoids and those of a DMBA-treated organoid-derived tumor to DMBA-untreated control organoids each.

### Animals

In the present study, a total of 14 BALB/c-*nu*/*nu* female mice (5 weeks of age; CLEA Japan) were used to confirm the tumorigenicity of DMBA-treated organoids, and to evaluate the effect of subcutaneous implantation/passaging of the induced tumors. The mice (*n* ≤ 5 per cage) were housed in plastic cages filled with recycled paper bedding (Paper-clean; Japan SLC, Inc., Hamamatsu, Japan) in an air-conditioned animal room maintained at 22°C (fluctuation range, within 1°C) and 55% relative humidity (fluctuation range, within 10%), on a 12:12-h light-dark cycle, and with free access to a standard chow diet CE2 (CLEA Japan). Mouse experiments were carried out according to the institutional guidelines and following the approval of the National Cancer Center Animal Ethics Committee of Japan.

### Tumorigenicity Assay in Nude Mice

*In vitro* DMBA-treated organoids, which were harvested and cryopreserved as described in a previous study (Naruse et al., 2020), were re-cultured for confirmation of tumorigenicity after injection into the nude mouse subcutis and for the evaluation of any histological and genetical alterations affecting the induced organoid-derived tumor tissues by passaging into other nude mice (Supplementary Figure S1B). The DMBA-treated organoids grown in four wells of a 12-well plate for each treatment condition (either 0 or 0.6 µM DMBA) were used for the tumorigenicity assays in nude mice. The organoids were resuspended in 40 µl medium and mixed with Matrigel at a 1:1 ratio, followed by injection into the right and left sides of the dorsal skin of female nude mice under isoflurane anesthesia (Zoetis Japan, Tokyo, Japan). A total of four injection sites in two mice were set up for each DMBA concentration. During the observation period until the next passage, the length and width of each subcutaneous nodule/Matrigel plug were measured using a caliper and the volumes were calculated as follows: Volume = length × (width)^2^ × 1/2. After 8 weeks, nude mice were euthanized under isoflurane anesthesia, and subcutaneous nodules or residual Matrigel plugs from the injected sites were excised. Each nodule/plug was cut into ∼8 mm^3^ cubes and implanted into a total of four sites in two mice per group. In the second and third passages, each subcutaneous nodule/Matrigel plug was similarly cut and implanted into a total of four sites in two mice per group; however, implantation was not performed at the third passage for the DMBA-untreated group. The residual tissues of each nodule/plug were fixed with 10% neutral buffered formalin, and several nodule/plug pieces were frozen in liquid nitrogen. If nodules accounted for more than 10% of the mouse body weight or the condition of the mice became poor, they were euthanized and the subcutaneous nodules were excised. These samples were preserved similarly to those collected from mice that were continuously observed until week 8.

### Histopathology and Immunohistochemistry

All subcutaneous nodules or residual Matrigel plugs fixed in 10% neutral buffered formalin were processed routinely and embedded in paraffin wax. Sections (of 3 μm thickness) were stained with hematoxylin and eosin (HE), and histopathologically evaluated. Paraffin-embedded sections were also used for immunohistochemistry analysis, and anti-human cytokeratin five mouse monoclonal (1:1,000 dilution; cat. no. 66727-2-Ig, Proteintech Group, Rosemont, IL, United States), anti-human cytokeratin 14 mouse monoclonal (1:400 dilution; clone LL002, Abcam, Cambridge, United Kingdom), anti-human cytokeratin 18 rabbit polyclonal (1:1,000 dilution; cat. no. 10830-1-AP; Proteintech), anti-mouse cytokeratin 19 rabbit monoclonal (1:200 dilution; clone EPNCIR127B, Abcam), anti-human α-smooth muscle actin (αSMA) rabbit monoclonal (1:1,000 dilution; clone EPR5368, Abcam), and anti-human estrogen receptor α (1:50 dilution, ERα, NCL-ER-6F11, Novocastra Laboratories, Newcastle upon Tyne, United Kingdom) primary antibodies were used. Antigen retrieval of sections was conducted in an autoclave at 121°C for 10 min in 10 mM citrate buffer (pH 6.0), except for cytokeratin 5 and cytokeratin 19 whereby 10 mM Tris-EDTA buffer (pH 9.0) was used. A polymer detection method (Histofine, MAX-PO, Nichirei Biosciences, Tokyo, Japan) was used to assess the expression and localization of the antigens, and the sections were lightly counterstained with hematoxylin for microscopic examination. Negative controls without primary antibodies were set using serial sections. Healthy murine mammary tissues and mammary adenocarcinomas with squamous cell differentiation were used as positive controls. In addition to these histological and immunohistochemical analyses, Sanger sequencing analysis was performed for the detection of mutations at *Vps13d* codon 295, *Tgfbr2* codon 549, and *Hras* colon 61.

### Quantitative Real-Time PCR and Western Blotting

1 μg total RNA for each sample (Isogen with Spin Column; NIPPON GENE, Tokyo, Japan) was prepared prior to reverse transcription to cDNA using Multiscribe Reverse Transcriptase with random primers (ThermoFisher Scientific). qRT-PCR was performed with SsoAdvanced Universal SYBR Green Supermix (BIO-RAD, California, United States) using CFX96 Touch (BIO-RAD). Reactions were run in triplicate for three subcutaneous nodules. Data were normalized with the housekeeping gene β-*Actin* and were calculated by the 2-ΔΔCT method (Livak and Schmittgen, 2001). The primer sequences were as follows: β-*Actin* forward 5′-AAG​TGT​GAC​GTT​GAC​ATC​CG -3′ and reverse GAT​CCA​CAT​CTG​CTG​GAA​GG-3′, *Vps13d* forward 5′-TGT​CGG​GAA​TGG​TGG​TAT​TT-3′ and reverse 5′-AGA​CAG​CAC​GCC​TCC​TTT​TA-3′, *Tgfbr2* forward 5′-GCA​TCC​AGA​TCG​TGT​GTG​AG-3′ and reverse 5′-CTC​ACA​CAC​GAT​CTG​GAT​GC-3’. For evaluation of stimulation in TGFβ-SMAD signalling pathway by *Tgfbr2* mutations, immunoblot analysis for SMAD2/3 and phospho-SMAD2 was conducted as follows. Twenty μg protein samples (EzRIPA Lysis buffer; ATTO, Tokyo, Japan) were subjected to SDS-polyacrylamide gel electrophoresis on 5–20% gradient acrylamide gels (ATTO), and the separated proteins were transferred to polyvinylidene difluoride membranes (Transblot Turbo System; Bio-Rad, Hercules, CA, United States). Immunoblotting was performed using anti-Smad2/3 rabbit polyclonal (1:1,000 dilution; #3102; Cell Signaling Technology, Danvers, MA, United States) and anti-phospho-Smad2 rabbit monoclonal (1:1,000 dilution; clone138D4, Cell Signaling), followed by exposure to peroxidase-labeled anti-rabbit polyclonal goat antibodies and the development of chemiluminescence signals with luminol (5-amino-1,2,3,4-tetrahydrophthalazine-1,4-dione; ATTO). Chemiluminescence was detected with LAS-3000 (Fujifilm, Tokyo, Japan). For detection of β-actin, 10 μg protein samples, a primary antibody (1:2,000 dilution; clone AC-15, Sigma-Aldrich), and peroxidase-labeled anti-mouse polyclonal rabbit antibody were used.

### Statistical Analysis

All quantitative data are presented as mean ± SD values. Volumes of subcutaneous residual Matrigel plugs and nodules were analyzed by the Student’s *t*-test following the *F*-test for homogeneity of variance in comparison between the DMBA-treated and control groups. When homogeneity of variance was not confirmed, Welch’s *t*-test was applied. With regards to the incidence of histopathological findings, the Fisher’s exact probability test was performed to compare the 0.6 μM DMBA-treated and negative control groups.

## Results

### Limited Numbers of DNA Mutations Were Detected in the Organoids After DMBA Treatment, but Increased Dramatically After Injection Into the Nude Mouse Subcutis

**FIGURE 1:**
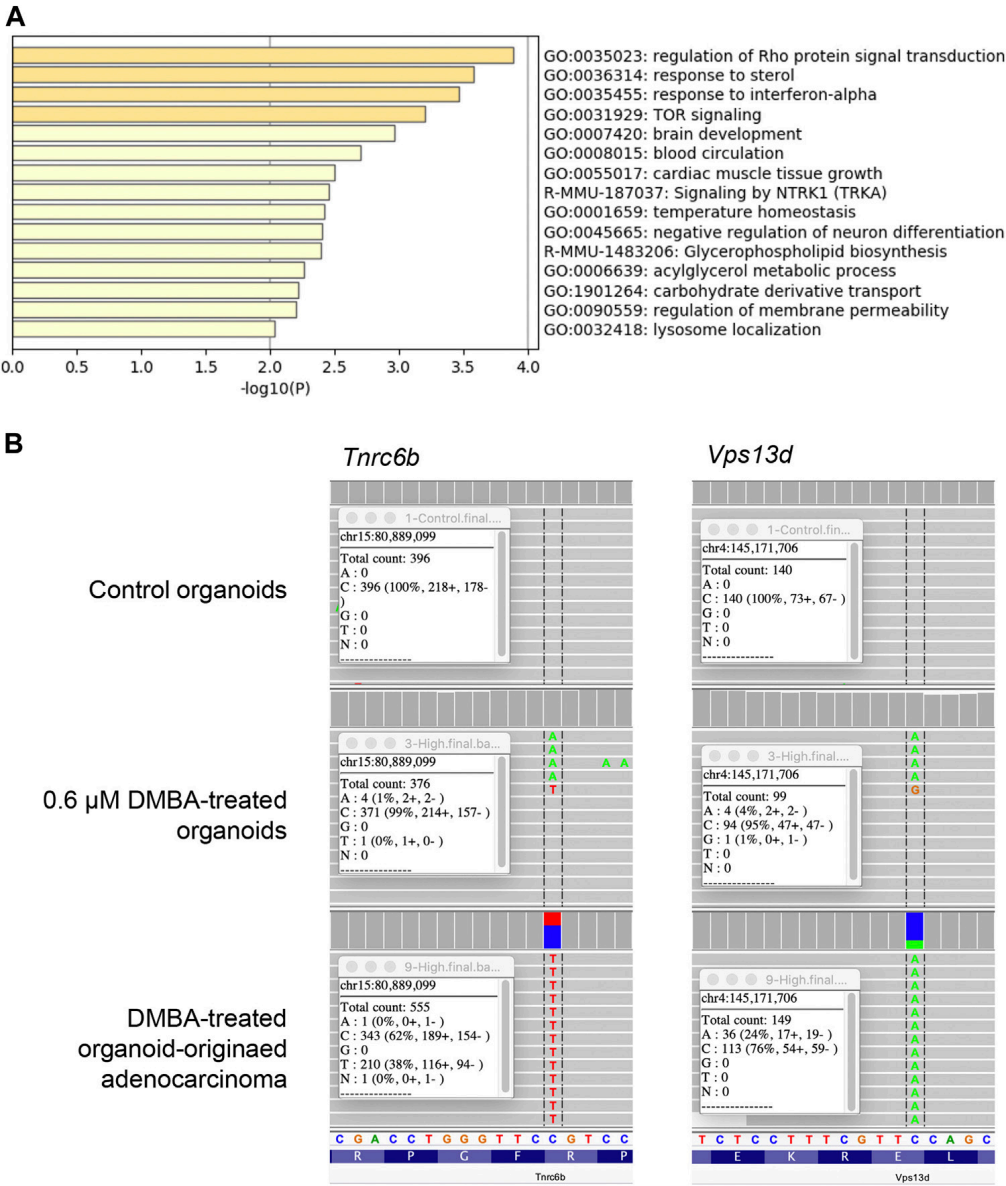
**(A)** Gene ontology clusters of single nucleotide variants identified by WES in a DMBA-treated organoid-derived subcutaneous adenocarcinoma. **(B)** A chart of *Vps13d* ^E295*^ and *Tnrc6b* ^R1149C^ mutations, obtained using the Integrative Genomics Viewer. These mutations were observed in both the DMBA-treated organoids and the DMBA-treated organoid-derived subcutaneous adenocarcinomas.

To evaluate the genetic alterations associated with DMBA-induced carcinogenesis in mammary tissue-derived organoids after injection into the subcutis of nude mice, WES analysis was conducted on the 0.6 μM DMBA-treated organoids and DMBA-treated organoid-derived subcutaneous adenocarcinoma tissue in comparison with the DMBA-untreated organoid negative control. The DMBA-induced gene mutations were selected for by removing shared single nucleotide variants (SNVs) in the three types of experimental sample (0.6 μM DMBA-treated organoids, the DMBA-induced organoid-derived adenocarcinoma tissues, and negative control organoids), after mapping and variant calling relative to the GRCm38/mm10 mouse reference genome. A total of 414 and 142 SNVs were identified in the 0.6 μM DMBA-treated organoids and the DMBA-induced adenocarcinomas, respectively (Supplementary Figure S2A). DMBA-induced organoid-derived adenocarcinoma SNVs included gene ontology clusters implicated in the regulation of Rho protein signal transduction, and the sterol and interferon-α responses (Figure 1A, Supplementary Table S1). Of note, SNVs shared between the DMBA-induced organoid-derived adenocarcinomas and 0.6 μM DMBA-treated organoids (prior to injection into the subcutis of nude mice) were limited to 10 genes, including *Tnrc6b* and *Vps13d*, which were selected by the IGV (Figure 1B).

### A Unique Mutational Pattern Was Observed in the Organoids After DMBA Treatment, and the Organoid-Derived Adenocarcinomas After Injection Into the Subcutis of Nude Mice

**FIGURE 2:**
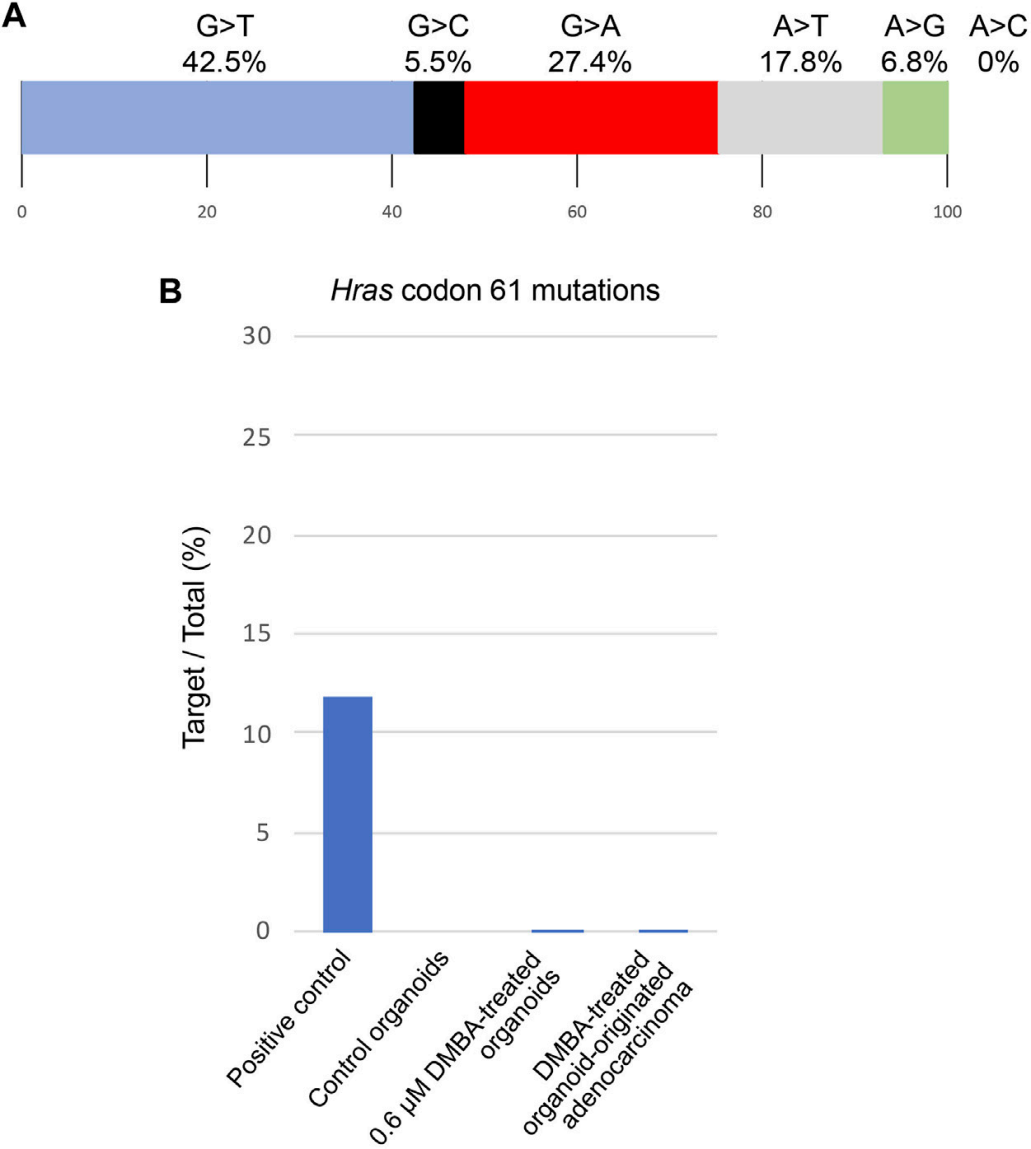
**(A)** Frequencies of the different nonsynonymous single nucleotide substitutions found in a DMBA-induced organoid-derived subcutaneous adenocarcinoma. **(B)** Absolute quantification of mutations at *Hras* codon 61 using a 3D Digital PCR System.

**FIGURE 3:**
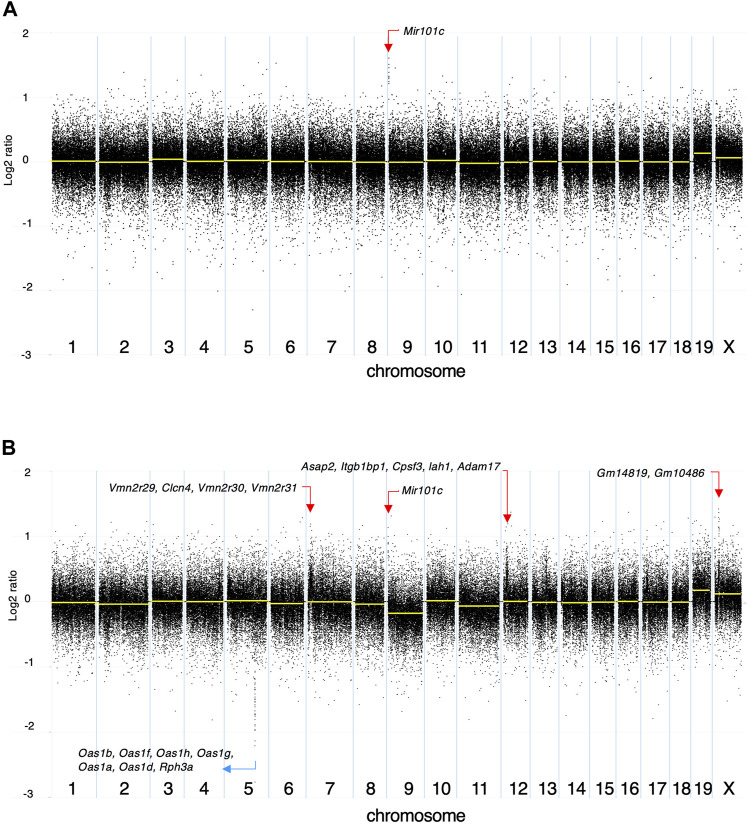
Copy number alteration (CNA) analysis. The log2 ratios of **(A)** DMBA-treated organoids to DMBA-untreated control organoids and **(B)** a DMBA-treated organoid-derived tumor to DMBA-untreated control organoids each. Each dot represents a genomic coordinate (*X*-axis) and Log2 ratio (*Y*-axis: compared with negative control). Large-scale gains/deletions as a shift of the dots and segmental gains/deletions were observed on several chromosomes.

Among the 142 SNVs detected in the DMBA-induced organoid-derived adenocarcinoma, guanine (G)-to-thymine (T) transversions, and G to adenine (A) transitions were most prevalent (Figure 2A). This mutational pattern differed from the typical mutational patterns (predominantly A to T transversions) induced by *in vivo* DMBA-treatment. The SNVs with G:C to T:A transversion- or G:C to A:T transition-containing genes included cancer-associated genes such as *Tgfbr2* and *Tusc3* (Korkut et al., 2018; Vasickova et al., 2018). In addition, the *Hras* codon 61 mutations did not appear amongst the 142 SNVs (Supplementary Figure S2B). Absolute quantification of mutations at *Hras* codon 61 was determined using a 3D Digital PCR System, and *Hras* mutational frequency was below the detection limit of the digital PCR system (0.1%) not only in DMBA-treated organoids, but also in the organoid-derived DMBA-induced adenocarcinomas in the nude mouse subcutis (Figure 2B). To determine the extent of chromosomal aberrations in DMBA-treated organoids and DMBA-treated organoid-derived subcutaneous adenocarcinomas, CNA analysis was conducted. Gains as a shift of the dots on chromosome 19, and segmental gains on chromosome 9 were observed in the both DMBA-treated organoids and DMBA-treated organoid-derived subcutaneous adenocarcinomas (Figures 3A,B). Other gains as a shift of the dots on chromosome X and deletions on chromosome 9, as well as segmental gains on chromosome 7, 9, 12, and segmental deletion on chromosome 5 were observed in the DMBA-treated organoid-derived adenocarcinomas. The locus with segmental gains included several cancer-associated genes, e.g., *Asap2* and *Adam17*, in the DMBA-treated organoid-derived adenocarcinomas (Figure 3B).

### The DMBA-Induced Organoid-Derived Tumor Tissues Were Low-Grade Carcinomas, Mainly Differentiated Into Luminal Lineages, and Exhibiting Partial Squamous Cell Differentiation

**FIGURE 4:**
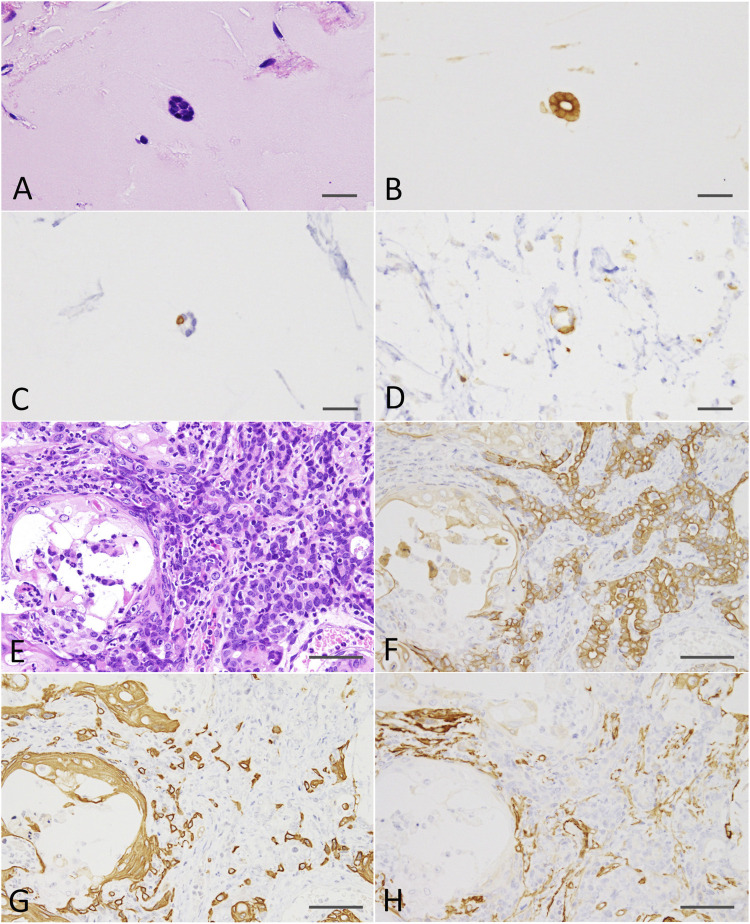
**(A)** A ductule-like structure in the residual Matrigel plug of the negative control. HE staining; scale bar, 20 μm. **(B)** Glandular marker CK18-positive ductule-like structures. **(C)** A basal/myoepithelial marker, CK14-positive epithelial cells were scattered in the ductule-like structures. **(D)** Several ductule-like structures were surrounded by αSMA-positive myoepithelial cells. **(E)** A low-grade adenocarcinoma with squamous cell differentiation **(left side)** induced by the injection of DMBA-treated organoids into the nude mouse subcutis. HE staining; scale bar, 50 μm. **(F)** Adenocarcinoma cells were positive for CK18. **(G)** Adenocarcinomas were partly positive for CK14 and squamous cell differentiated regions were positive for CK14. **(H)** Adenocarcinomas were partly positive for αSMA. Note; interstitial cells including blood vessels were also positive for αSMA. CK, cytokeratin; αSMA, α smooth muscle actin.

At the end of the experiment (on week 8 after DMBA-treated organoid injection into the subcutis of nude mice), residual Matrigel plugs were macroscopically observed as small (average volume and s.d., 32.5 ± 5.9 mm^3^) and clear fragments in the DMBA-untreated group (negative control; 4 of 4 injection sites), or opaque white nodules (4 of 4 injection sites; as above, 80.9 ± 21.8 mm^3^; *p* < 0.01 vs control; Supplementary Figure S3) with slight skin ulceration (1 of 4 injection sites) in the 0.6 μM DMBA treatment group. Histopathology analysis revealed the presence of scattered small ductule-like structures in the residual Matrigel plugs of the control samples (Figure 4A). In contrast, the DMBA-induced organoid-derived tumor tissues (4 of 4 injection sites; *p* < 0.05) resembled low-grade adenocarcinomas with double or multiple layered glandular structures, and partially showed squamous cell differentiation accompanied with severe neutrophil infiltration (Figure 4E), as observed in our previous study (Naruse et al., 2020). Invasive appearance to surrounding tissues, e.g., cutaneous muscle of nude mice, and the epithelial cells with some nuclear abnormalities, for example enlargement, clouding and prominent nucleoli in the tumor tissues, indicated the tumors to be aggressive in nature (Supplementary Figure S4A). Our immunohistochemistry results showed that all the small ductule-like epithelia in the control samples were glandular cytokeratin (CK) 18/CK19-positive, interspersed with basal/myoepithelial CK14-positive cells. Several ductule-like structures were surrounded by α smooth muscle actin (αSMA)-positive myoepithelial cells (Figures 4B–D). The DMBA-induced organoid-derived carcinomas contained both CK18/CK19-positive cells and CK14/αSMA-positive cells (Figures 4F–H), and CK5 showed negative (data not shown). The carcinomas (3 of 4 injection sites) were partly positive for ERα (Supplementary Figure S4B). The small ductule-like organoids and organoid-derived carcinomas were negative for CK5 and ER-α (data not shown).

### DMBA-Induced Low-Grade Mammary Adenocarcinoma Progressed to SCC by Passaging Into the Subcutis of Nude Mice

**FIGURE 5:**
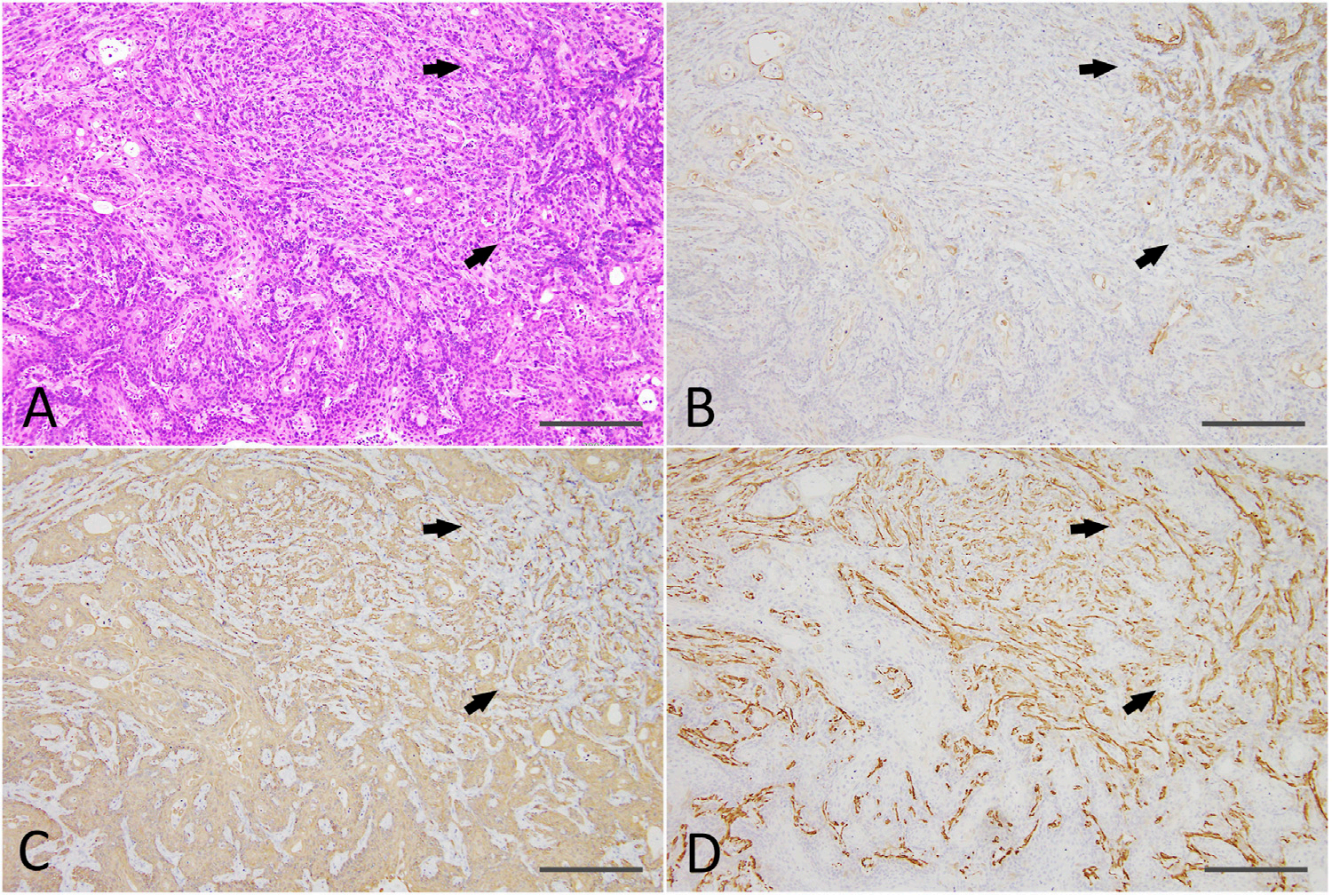
**(A)** A SCC **(lower half and left side)** with a residual low-grade adenocarcinoma region **(right upper;** arrows) and an intermediate region **(upper middle)** after the third passage of a DMBA-treated organoid-derived tumor into the nude mouse subcutis. HE staining; scale bar, 200 μm. **(B)** The residual low-grade adenocarcinoma region was positive for CK18 (arrows). **(C)** The SCC was positive for CK14, and the residual adenocarcinoma (arrows) and intermediate region was also partly positive for CK14. **(D)** The residual adenocarcinoma (arrows) and intermediate region was positive for αSMA. Note: αSMA—positive cells in the SCC were interstitial fibroblasts. SCC, squamous cell carcinoma; CK, cytokeratin; αSMA, α smooth muscle actin. SCC, squamous cell carcinoma.

Residual Matrigel plugs transplanted into other nude mice during passages 1 and 2 did not grow (a total of 8/8 injection sites), and residual Matrigel plugs were not transplanted at passage 3 into the control group. In contrast, transplanted tumors grew rapidly at passages 1–3 in the 0.6 μM DMBA treatment group, as compared to not only each corresponding control (residual Matrigel plugs), but also to those of the first injected organoids at passage 0 at each time point (Supplementary Figure S3). At passages 2–3 and the final sampling, excised residual Matrigel plugs were macroscopically observed as clear/white fragments (average volume and s.d., 12.1 ± 7.0 mm^3^) in the controls (8 injection sites), or yellow/white neoplastic nodules (as above, 1,264.2 ± 1,044.4 mm^3^; *p* < 0.01) in the 0.6 μM DMBA treatment group (12 injection sites). Histopathology analysis revealed a few small ductule-like epithelial structures in the Matrigel residue at passages 1–2 of the control samples. In the 0.6 μM DMBA treatment group, low-grade adenocarcinomas with evidence of squamous cell differentiation were retained (1 of 4 transplanted sites) or partly progressed to squamous cell carcinomas (SCCs; 3 of 4 transplanted sites) at passage 1, and were partly replaced by SCCs at passages 2–3 (Supplementary Figure S4C); however, low-grade adenocarcinoma regions remained even after the third passage (Figure 5A). Immunohistochemistry data showed that SCCs were positive for CK5/CK14, partly positive for ERα (3 of 9 injection sites examined) (Supplementary Figure S4D) and negative for αSMA and CK18/CK19 (Figures 5B–D).

### The Principle DMBA-Induced Mutations Were Maintained Even After Progression to SCC

The mutations at *Vps13d* codon 295 and *Tgfbr2* codon 549, found in the DMBA-treated organoid-derived adenocarcinomas were observed also in the passaged nodules, diagnosed as SCCs (Supplementary Figures S5A,B). As for *Tgfbr2*, the mutation rates in the SCCs appeared to increase as compared to those detected in the DMBA-treated organoid-derived adenocarcinomas. In addition, the absence of *Hras* codon 61 mutations was confirmed in all of the passaged nodules by Sanger sequencing (Supplementary Figure S5C). To evaluate the effects of mutations at *Vps13d* codon 295 and *Tgfbr2* codon 549, quantitative real-time PCR for *Vps13d* and *Tgfbr2*, and immunoblot analysis for SMAD2/3, which was a transcription factor in TGFβ-SMAD signalling pathway, were conducted using the DMBA-treated organoid-derived adenocarcinomas, with reference to normal mammary tissues and mammary adenocarcinomas obtained in an *in vivo* DMBA-treated experiment using BALB/c-*Trp53* knockout female mice (Machida and Imai, 2021). The gene expression levels of both *Vps13d* and *Tgfbr2* were not affected by the presence of mutations (Supplementary Figure S6A). In contrast, immunoblots for SMAD2/3 and phospho-SMAD2 indicated stimulations in TGFβ-SMAD signalling pathway in relation to the *Tgfbr2* mutations (Supplementary Figure S6B).

The mutations at *Vps13d* codon 295 and *Tgfbr2* codon 549 were not observed in adenocarcinoma samples (8 of 8 examined, data not shown) obtained in an *in vivo* DMBA-treated experiment using BALB/c-*Trp53* knockout female mice (Machida and Imai, 2021).

## Discussion

In the present study, we reported that the *in vitro* treatment of healthy mammary tissue-derived organoids of heterozygous BALB/c-*Trp53* knockout mice with 0.6 μM DMBA induced tumors after injection into the subcutis of nude mice. The purpose of this work was to validate whether the organoid-based carcinogenesis model was genetically and/or histologically similar to the DMBA-induced *in vivo* carcinogenesis system. To this end, we first analyzed gene mutations by WES in the 0.6 μM DMBA-treated organoids prior to injection into nude mice, and compared them to DMBA-untreated organoids (negative controls). Our result showed that SNVs were detected in 10 genes (including *Vps13d* ^E295*^ and *Tnrc6b* ^R1149C^) in the 0.6 μM DMBA-treated organoids prior to injection into the subcutis of nude mice. Although no clear associations between mutations in *Vps13d* and human cancer have been documented, VPS13D was found to play a dual role in mitochondrial morphology and peroxisome biogenesis in human cells (Baldwin et al., 2021). It has been reported that VPS13D mutants lacking a ubiquitin-associated (UBA) domain exhibit defects in the mitochondrial size and clearance and exhibit semi-lethality (Anding et al., 2018). According to the catalogue of somatic mutations in cancer (COSMIC) database v94, mutation incidence in skin and breast cancer are 16.37 and 6.90, respectively (https://cancer.sanger.ac.uk/cosmic/gene/analysis?ln=VPS13D). As for *Tnrc6b*, an integrated genomic analysis for human hepatocellular carcinoma using MutSig algorithms identified several novel driver genes, including *TNRC6B* (Li et al., 2018). Furthermore, the COSMIC database v94 revealed the mutation incidence in liver, skin, and breast cancer to be 8.62, 8.61, and 4.54, respectively, suggesting a possibility that the mutations in *Vps13d* and/or *Tnrc6b* may be associated with the carcinogenesis demonstrated in the present study.

We next analyzed SNVs by WES in a DMBA-treated organoid-derived subcutaneous adenocarcinoma after injection into the subcutis of nude mice, with reference to the DMBA-untreated organoids. Of note, SNV number increased dramatically from 10 in the DMBA-treated organoids to 142 in the tumor after injection into nude mice. Moreover, the SNV-containing genes included several cancer-associated ones such as *Tusc3* ^R18H^ (Vasickova et al., 2018) and *Tgfbr2* ^D549Y^ (Korkut et al., 2018). This finding showed that the clonal expansion of carcinoma cells following injection into the subcutis of nude mice harbored multiple mutated cancer-associated genes. In addition, our analysis of the mutational patterns present in the SNVs revealed that G-to-T transversions and G-to-A transitions were most common, which was in contrast to the typical mutational patterns (predominantly A-to-T transversions) previously reported following DMBA treatment *in vivo* (Nassar et al., 2015). Stable DNA adducts formed by DMBA treatment, which were considered to be directly associated with DMBA-induced gene mutations, were previously identified and quantitated. 7-Methylbenz[*a*]anthracene (MBA)-12-CH_2_-N7adenine and 7-MBA-12-CH_2_-N7guanine were predominant in DMBA-treated *in vivo* studies, at frequencies of 39 and 13%, respectively, in rat mammary tissue (Todorovic et al., 1997), and 79 and 20%, respectively, in mouse skin (Devanesan et al., 1993). DMBA-DNA adduct formation *in vitro* using 3-methyl-cholanthrene-induced rat liver microsomes was also examined, and 7-MBA-12-CH_2_-N7adenine (82%) and 7-MBA-12-CH_2_-N7guanine (17%) were identified (RamaKrishna et al., 1992). Moreover, these DMBA-DNA adduct data were consistent with the typical mutational patterns (predominantly A-to-T transversions) induced by *in vivo* DMBA treatment. Meanwhile, Nassar et al. reported that a comparison of the mutational patterns of DMBA-induced dermal SCCs with their lung or lymph node metastases, revealed that metastasis-specific mutations were primarily G-to-A transitions and G-to-T transversions, indicating that they were DMBA-independent in origin. Based on these previous findings, the DMBA-targeted genes and DMBA-induced substitution patterns observed in the organoids used in the present study were found to be DMBA-specific. These genetic mutations then underwent selective pressure in the present culture conditions, similarly to the *in vivo* tumor metastatic process.

Moreover, *Hras* codon 61 mutations, which were found at high frequencies in mammary adenocarcinomas induced by oral DMBA treatment of the heterozygous BALB/c-*Trp53* knockout mice, did not appear in the list of 142 SNVs. To detect low-frequency mutants, the absolute quantification of mutations at *Hras* codon 61 was performed using a 3D Digital PCR System. However, no mutations at *Hras* codon 61 were confirmed in both the 0.6 μM DMBA-treated organoids and the DMBA-treated organoid-derived adenocarcinomas in the subcutis of nude mice using this technique. We also analyzed the presence or absence of mutations at *Hras* codon 61 by Sanger sequencing in progressed SCCs obtained via the passage of DMBA-treated organoid-derived tumors to the subcutis of nude mice. This technique did not identify any mutations either, indicating that *Hras* codon 61 mutations were not associated with mammary carcinogenesis in the present study. On the other hand, gene mutations at *Vps13d* codon 295 and *Tgfbr2* codon 549, which were found in DMBA-treated organoids and/or the DMBA-treated organoid-derived adenocarcinomas were not observed in mammary adenocarcinomas induced by oral DMBA treatment of the heterozygous BALB/c-*Trp53* knockout mice. Therefore, the *Hras* codon 61 mutations were suggested to act as a driver in the orally DMBA-treated mouse model. In contrast, mutations in other genes were speculated to be as drives in DMBA-treated organoid-derived adenocarcinomas.

CNA analysis revealed several large-scale gains/deletions and various segmental gains/deletions particularly in the DMBA-treated organoid-derived subcutaneous adenocarcinomas after injection into the subcutis of nude mice. The locus with segmental gains included several cancer-associated genes, e.g., *Asap2* (Fujii et al., 2021) and *Adam17* (Shen et al., 2016). In addition to the SNVs, the CNAs were considered to be associated with the present DMBA-induced mammary carcinogenesis, and comprehensive approaches should be needed (Liu et al., 2020) to understand the mechanisms of mammary carcinogenesis induced by not only DMBA, but also other chemical carcinogens.

We further evaluated the histopathological and immunohistochemical characteristics of DMBA-treated organoid- low-grade adenocarcinomas passaged to other nude mice, to examine whether they progressed to high-grade adenocarcinomas (characterized by the presence of biphasic structures differentiated into luminal and myoepithelial lineages), as observed in the DMBA-induced *in vivo* carcinogenesis study (Machida and Imai, 2021).

Our histopathology analysis showed the presence of small ductule-like epithelial structures in the Matrigel plugs after the injection of DMBA-untreated organoids (and up to the second passage) in the control mouse group. The immunohistochemistry data revealed the presence of CK18/CK19-positive epithelial cells in the ductule-like structures, indicating that the epithelial cells were mainly of luminal cell origin (Bocker et al., 2002). At the same time, the coexistence of CK14/αSMA-positive basal/myoepithelial cells was observed in the mouse mammary tissue-derived organoids. In the 0.6 μM DMBA-treated group, low-grade adenocarcinomas, containing luminal CK18/CK19-positive or basal/myoepithelial CK14/αSMA-positive cells, developed after injection of the organoids into the nude mouse subcutis. This indicated that both the CK18/CK19-positive luminal cells and the CK14-positive basal/myoepithelial cells were genetically altered by *in vitro* DMBA treatment. When the low-grade adenocarcinomas were passaged into other nude mice, they did not transform into high-grade lesions displaying biphasic structures, and retained their original characteristics to some extent, even after the third passage. On the contrary, the adenocarcinomas partly transformed into SCCs or were replaced by SCCs during the passages into mice. The resulting SCCs were positive for CK5/CK14 and negative for αSMA and CK18/CK19. These results suggested that CK5/CK14-positive basal cells with DMBA-induced DNA mutations showed clonal expansion in the nude mouse subcutis during passage. In human breast tissue, CK5-positive cells act as precursors or committed stem cells, responsible for the regeneration of adult glandular or myoepithelial cells (Bocker et al., 2002). On the other hand, in human breast cancer cases, CK18/19-positive luminal cells are predominant, and it was speculated that cancer cells originate from a late stage of the glandular epithelial differentiation pathway (Bocker et al., 2002). At the same time, CK5/CK14 expression was observed in a subgroup of estrogen receptor-negative tumors, and CK5/CK14-positive sporadic breast cancers with an incidence of ∼9% were shown to arise from glandular committed progenitor cells rather than from true CK18-negative basal cells. This is in contrast to cells derived from true basal phenotypic cells as is the case in heritable *BRCA1*-mutated breast cancers (Laakso et al., 2005). This was considered to be associated with a function of wild-type BRCA1, which acts as a stem cell regulator and promoter of differentiation into the glandular epithelium of normal breast tissue (Foulkes, 2004). Although *Brca1* SNVs were not found in the present study, SNVs targeting other genes (e.g., *Tgfbr2*), which are implicated in squamous cell homeostasis (Guasch et al., 2007), may be associated with the clonal expansion of CK5/CK14-positive basal cells during carcinogenesis. The mutation rates of *Tgfbr2* in the progressed SCCs appeared to increase as compared those detected in the DMBA-treated organoid-derived adenocarcinomas observed in the present study, suggesting an association between squamous cell differentiation and the function of TGFBR2. The mutations found in the DMBA-treated organoid-derived adenocarcinomas and the absence of *Hras* codon 61 mutations were maintained in the subsequent SCCs. These observations further support the existence of genetically-clonal lineage relationship between the 0.6 μM DMBA-treated organoids, the organoid-derived adenocarcinomas developed after injection into the nude mouse subcutis, and the SCCs progressed by passaging. Causes of the unique genetic and histological characteristics of DMBA-induced mammary tumors in the present organoid-based carcinogenesis model were not clear. However, cell metabolic functions have been reported to be well represented in three-dimensional (3D) culture conditions, e.g., spheroid and organoid culturing, as compared to 2D culturing (Cannon et al., 2017). In addition, chemical carcinogens were directly exposed to targeted epithelial cells *in vitro*, regardless of their absorption, distribution and excretion arose in animal models, and these factors should at least partly relate to the DMBA-induced genetic and histological characteristics in the present organoid-based model.

In summary, DMBA exhibited carcinogenic abilities in an organoid-based *ex vivo* mammary carcinogenesis model. However, the organoid-derived tumors were histologically low-grade adenocarcinomas and progressed to SCCs only after passage into the subcutis of nude mice. This is in contrast to the high-grade adenocarcinomas, which were observed in an *in vivo* DMBA-induced mammary carcinogenesis model, generated using the same mouse strain. In addition, the most common mutational patterns observed in the DMBA-treated organoid-derived tumors were G:C to T:A transversions and G:C to A:T transitions, accompanied by an absence of mutations at *Hras* codon 61, which is different from the typical mutation characteristics observed in *in vivo* DMBA-induced carcinogenesis models. As a possible mechanism to account for the differences in the histological and genetic characteristics between the *ex vivo* and *in vivo* models, we propose that the DMBA-induced gene mutations were likely to have undergone selective pressure in the present organoid culture conditions. The mutated cells could then have expanded clonally in the mouse subcutis and targeted alternative genes, partly affecting the histology of the induced tumors. Although mutations at *Hras* codon 61, which is not a major driver of human breast cancer, were not detected, other cancer-associated genes such as *Tusc3* and *Tgfbr2*, were found to be mutated in the present study. Therefore, further studies are needed to clarify whether organoid-based carcinogenesis models generated following chemical treatment *in vitro* could be applied to the detection of early genetic events, leading to the clarification of the novel mode of action of chemical carcinogenesis.

## Data Availability

The datasets presented in this study can be found in online repositories. The names of the repository/repositories and accession number(s) can be found below: DDBJ database: https://ddbj.nig.ac.jp/resource/bioproject/PRJDB12224, submission no: DRA012805, accession number BioProject: PRJDB12224.

## Supplementary Material

Supplementary Figure S1 An overview of the samples used in the present study. **(A)** Organoids were cultured from normal mammary tissues of heterogeneous BALB/c-*Trp53* knockout female mice, treated three times with 0.2 or 0.6 µM for 24 hours, or left untreated, and injected subcutaneously into the subcutis of nude mice as described in our previous study. **(B)** In the present study, genomic analyses were conducted using DMBA-treated cryopreserved organoid and subcutaneous tumor samples. In addition, cryopreserved organoids were re-cultured and injected into the nude mouse subcutis. The resulting tumors were passaged to other nude mice, and the genomic and histological analysis were performed using these subcutaneous tumors.

Supplementary Figure S2
**(A)** Numbers of SNVs identified by WES in the DMBA-treated organoids and a DMBA-treated organoid-derived subcutaneous adenocarcinoma. **(B)** A list of *Hras* codon 61 mutations obtained using the Integrative Genomics Viewer. No *Hras* codon 61 mutations were observed in the negative control, the 0.6 μM DMBA-treated organoids, or the DMBA-treated organoid-derived subcutaneous adenocarcinoma. DMBA, 7,12-dimethylbenz[*a*]anthracene; SNVs, single nucleotide variants; WES, whole exome sequence analysis.

Supplementary Figure S3 Growth curves of transplanted tumor tissues. Closed symbol, control; open symbol, 0.6 μM DMBA. Circle, P0; triangle, P1; square, P2; diamond, P3. ***p* < 0.01, **p* < 0.05 vs. corresponding controls. ^a^ *p* < 0.001, ^b^ *p* < 0.01, ^c^ *p* < 0.05 vs. *p* 0–0.6 μM DMBA. P0, original adenocarcinomas induced after subcutaneous injection of DMBA-treated organoids. P1–P3, 1st passage to 3rd passage of DMBA-induced adenocarcinomas implanted into other nude mice.

Supplementary Figure S4
**(A)** An adenocarcinoma with invasive growth to cutaneous muscle of nude mice (arrows) induced by the injection of DMBA-treated organoids into the nude mouse subcutis. Some nuclear abnormalities, for example enlargement, clouding and prominent nucleoli, were observed in the carcinoma cells. HE staining; scale bar, 50 μm. **(B)** A serial section of **(A)**. Adenocarcinoma cells were partly positive for ERα. **(C)** A SCC after the third passage of a DMBA-treated organoid-derived tumor into the nude mouse subcutis. HE staining; scale bar, 50 μm. **(D)** A serial section of **(C)**. SCC cells were partly positive for ERα. DMBA, 7,12-dimethylbenz[*a*]anthracene; SCC, squamous cell carcinoma; ERα, estrogen receptor α.

Supplementary Figure S5 The principle mutations in the DMBA-treated organoids-derived tumors, as confirmed by Sanger sequencing. **(A)** Mutations at *Vps13d* codon 295. The mutation rates appeared to be comparable throughout the tumor passaging. **(B)** Mutations at *Tgfbr2* codon 549. The mutation rates in the SCCs appeared to increase as compared to those detected in the DMBA-treated organoid-derived carcinomas. **(C)** No mutations were observed at *Hras* codon 61 in the adenocarcinomas and SCCs.

Supplementary Figure S6
**(A)** Expression levels of *Vps13d* (left) and *Tgfbr2* (right) in DMBA-treated organoid-derived tumors, evaluated with reference to normal mammary tissues and mammary adenocarcinomas obtained in an *in vivo* DMBA-treated experiment using BALB/c-*Trp53* knockout female mice (Machida and Imai, 2021). No difference in the expression levels related to the mutations in *Vps13d* and *Tgfbr2*. **(B)** Immunoblots for Smad2/3 and phospho-Smad 2 using DMBA-treated organoid-derived tumors with reference to normal mammary tissues and mammary adenocarcinomas obtained in an *in vivo* DMBA-treated experiment, indicating stimulations in TGFβ-SMAD signalling pathway in relation to the *Tgfbr2* mutations.
