# Supplementary material for: The Unique Genetic and Histological Characteristics of DMBA-Induced Mammary Tumors in an Organoid-Based Carcinogenesis Model
Source: Front Genet. 2021 Nov 29;12:765131. doi: 10.3389/fgene.2021.765131 (PMC8666664; doi:10.3389/fgene.2021.765131)
Supplement: Supplementary file 1 [file DataSheet1.pdf]

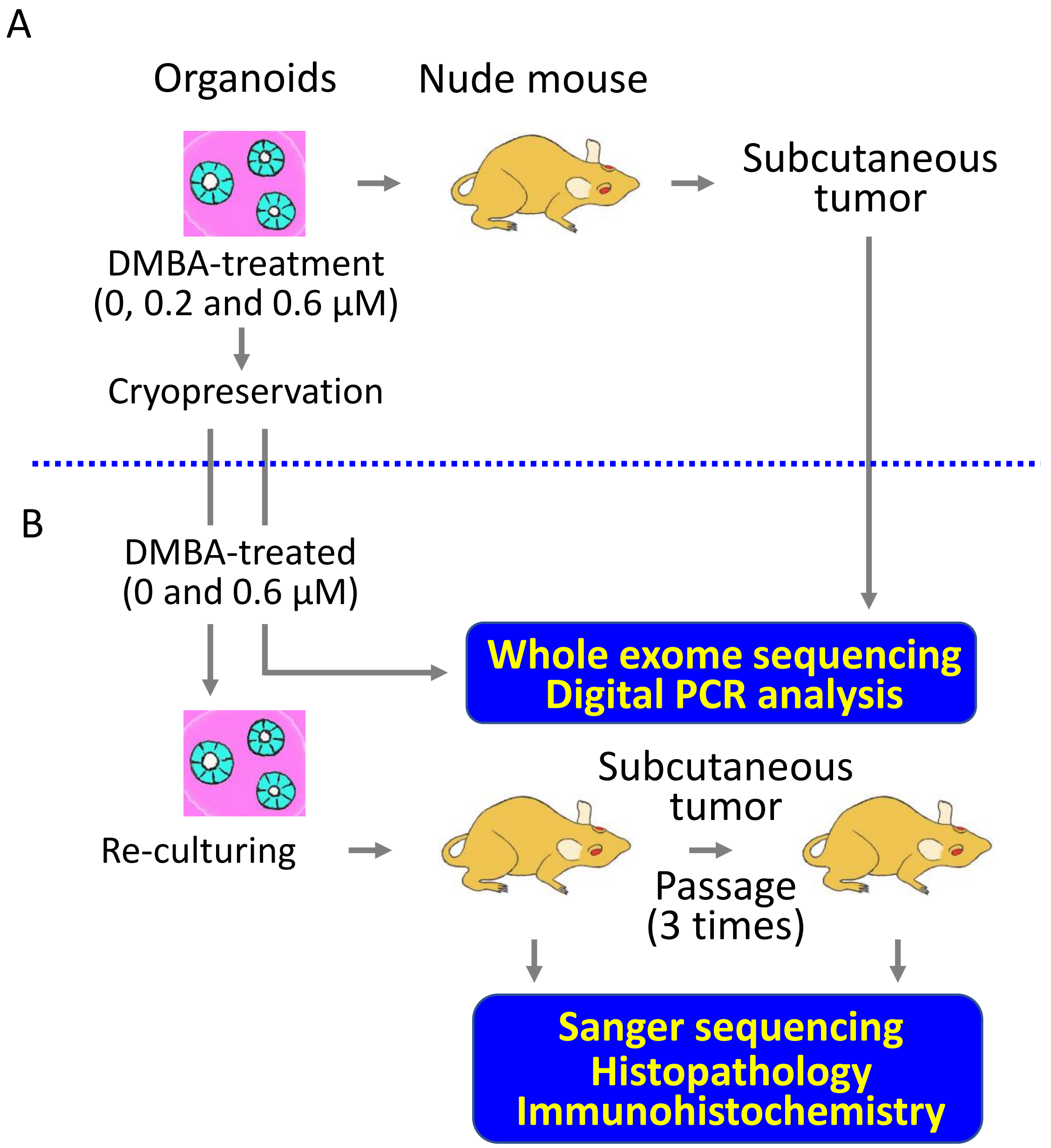

Supplementary Fig. S1

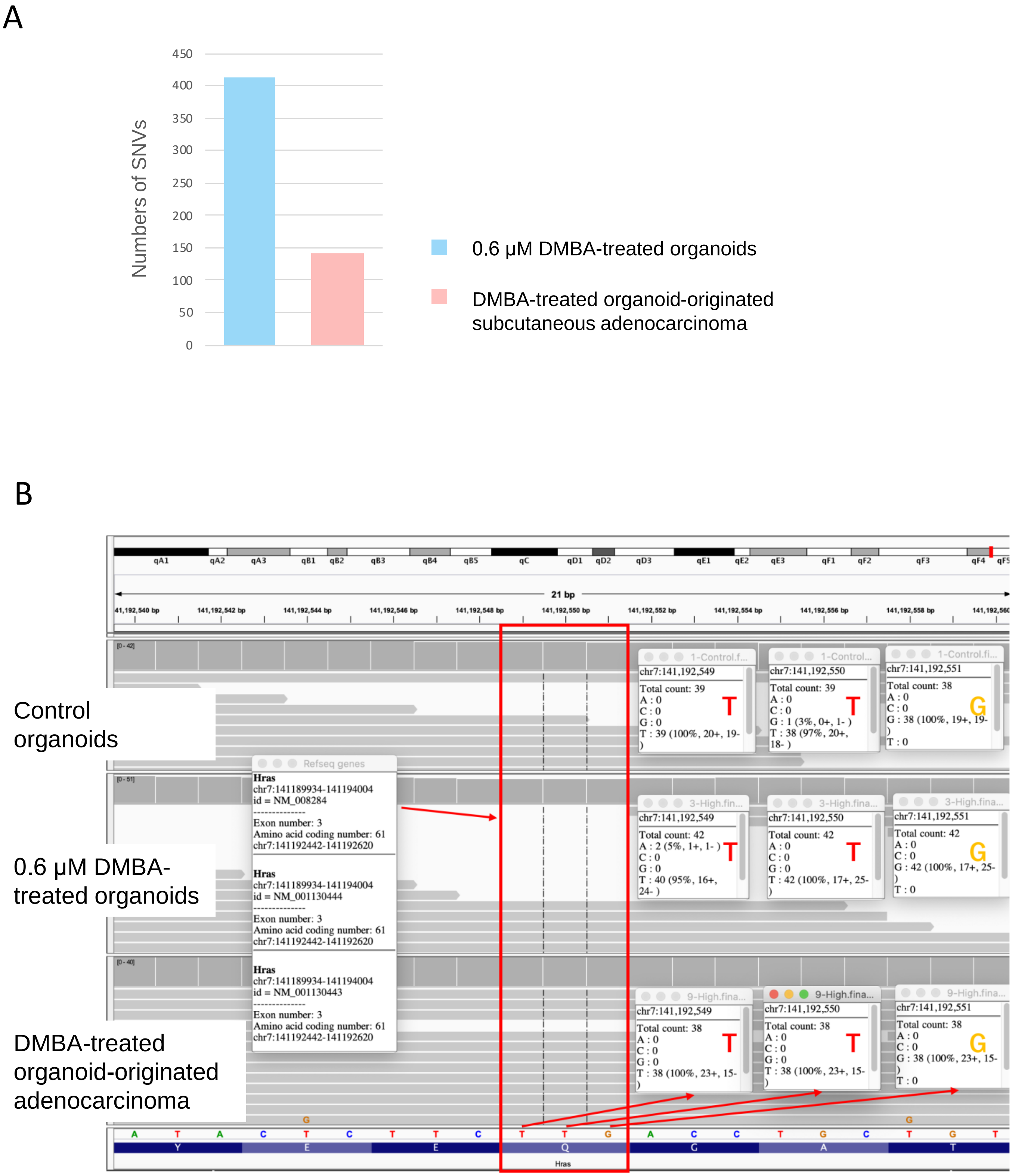

Supplementary Fig. S2

Supplementary Table S1

Single nucleotide variants in a DMBA-induced adenocarcinoma

(Colored 10 variants were observed also in DMBA-treated organoids)

CHROM	POS	REF	ALT	Func.refGene	Gene.refGene	ExonicFunc.refGene	mut freq	total reads
chr11	4893085	C	T	intronic	Nipsnap1	.	0.434	53
chr15	80889099	C	T	exonic	Tnrc6b	nonsynonymous_SNV	0.39	487
chr11	3242494	G	A	exonic	Eif4enif1	nonsynonymous_SNV	0.344	314
chr19	55198757	T	C	exonic	Gucy2g	nonsynonymous_SNV	0.303	66
chr2	69778079	G	A	exonic	Ccdc173	nonsynonymous_SNV	0.272	323
chr8	22012781	C	A	exonic	Atp7b	nonsynonymous_SNV	0.245	110
chr4	145171706	C	A	exonic	Vps13d	stopgain	0.242	149
chr10	3366821	C	T	exonic	Ppp1r14c	nonsynonymous_SNV	0.238	21
chr11	11943963	G	T	exonic	Grb10	synonymous_SNV	0.237	93
chr3	116883003	G	T	intronic	Frrs1	.	0.231	39
chr11	87404291	T	C	intronic	Rad51c	.	0.688	16
chr7	45628604	G	A	exonic	Rasip1	nonsynonymous_SNV	0.667	6
chr9	103365299	G	A	exonic	Cdv3	nonsynonymous_SNV	0.5	8
chr9	103365303	C	A	exonic	Cdv3	nonsynonymous_SNV	0.5	8
chr14	54988769	A	G	splicing	Myh7	.	0.5	12
chr17	7170404	G	T	exonic	Rps6ka2	nonsynonymous_SNV	0.5	8
chr8	121541794	G	T	UTR5	1700018B08Rik	.	0.444	9
chr9	103365343	A	T	exonic	Cdv3	nonsynonymous_SNV	0.444	9
chr9	103365294	G	T	exonic	Cdv3	nonsynonymous_SNV	0.429	7
chr2	122521922	A	G	exonic	Gm14085	nonsynonymous_SNV	0.401	152
chr2	27165555	C	A	exonic	Dbh	nonsynonymous_SNV	0.4	15
chr9	103365288	G	C	exonic	Cdv3	synonymous_SNV	0.4	5
chr2	153070865	G	A	exonic	Ccm2l	nonsynonymous_SNV	0.385	13
chr8	36146639	T	C	exonic	Prag1	nonsynonymous_SNV	0.375	16
chr9	116090512	C	A	exonic	Tgfbr2	nonsynonymous_SNV	0.367	30
chr11	61684906	G	A	exonic	Fam83g	nonsynonymous_SNV	0.364	11
chr17	28691853	G	T	exonic	Mapk14	synonymous_SNV	0.364	11
chr4	128786948	C	A	exonic	Zfp362	nonsynonymous_SNV	0.346	26
chr2	26021362	G	T	intronic	Ubac1	.	0.333	12
chr17	29367133	G	T	splicing	Fgd2	.	0.333	12
chr17	34600098	G	A	exonic	Ager	nonsynonymous_SNV	0.333	12
chr18	34840795	C	A	splicing	Reep2	.	0.333	12
chr13	95337174	T	C	UTR3	Zbed3	.	0.324	139
chr7	127538496	C	A	splicing	Srcap	.	0.308	13
chr7	143764498	C	A	UTR3	Mrgprg	.	0.308	13
chr8	13468390	G	T	intronic	Gas6	.	0.308	13
chr8	39006115	G	A	exonic	Tusc3	nonsynonymous_SNV	0.308	13
chr8	121785089	G	T	exonic	Jph3	nonsynonymous_SNV	0.308	13
chr17	35096351	C	T	exonic	Abhd16a	synonymous_SNV	0.308	13
chr19	8771302	T	A	exonic	Tmem223	synonymous_SNV	0.308	13
chr19	15984659	C	A	exonic	Cep78	nonsynonymous_SNV	0.308	13
chr9	45345090	G	T	intronic	Tmprss13	.	0.3	40
chr8	46209255	G	A	exonic	Slc25a4	nonsynonymous_SNV	0.294	17
chr10	86859765	G	T	exonic	Stab2	nonsynonymous_SNV	0.294	17
chr11	69060191	G	T	exonic	Borcs6	stopgain	0.294	17
chr4	32501632	T	A	exonic	Bach2	nonsynonymous_SNV	0.292	48
chr8	124528978	T	A	exonic	Cog2	nonsynonymous_SNV	0.292	281
chr19	30539614	G	A	ncRNA_exonic	Ppp1r2-ps3	.	0.286	14
chr17	35149347	G	A	exonic	Prrc2a	nonsynonymous_SNV	0.273	11
chr8	94500384	G	C	exonic	Nlrc5	nonsynonymous_SNV	0.265	34
chr14	32388355	T	A	UTR5	1700024G13Rik	.	0.263	19
chr15	76735363	C	A	exonic	Arhgap39	nonsynonymous_SNV	0.263	19
chr7	116170682	C	T	exonic	Plekha7	synonymous_SNV	0.25	8
chr11	3492035	G	T	intronic	Pla2g3	.	0.24	25
chr2	76050184	C	A	exonic	Pde11a	stopgain	0.235	34
chr11	68951094	C	T	exonic	Arhgef15	nonsynonymous_SNV	0.228	79
chrX	95926922	G	T	exonic	Zc3h12b	nonsynonymous_SNV	0.228	79
chr4	11076106	T	A	exonic	Ndufaf6	nonsynonymous_SNV	0.227	22
chr14	101729687	C	A	upstream	Lmo7	.	0.227	22
chr2	6100390	C	G	exonic	Proser2	nonsynonymous_SNV	0.222	18
chr19	8625641	G	A	exonic	Slc22a6	nonsynonymous_SNV	0.222	27
chr9	124126835	C	G	UTR3	Ccr5	.	0.221	312
chr7	25814995	A	G	exonic	Cyp2s1	nonsynonymous_SNV	0.216	37
chr19	25410338	G	T	exonic	Kank1	synonymous_SNV	0.215	65
chr11	58051098	A	T	exonic	Larp1	nonsynonymous_SNV	0.214	42
chr8	87781344	A	T	exonic	Zfp423	nonsynonymous_SNV	0.212	33
chr7	56191212	A	T	exonic	Herc2	synonymous_SNV	0.208	24
chr4	118381392	C	G	exonic	Szt2	nonsynonymous_SNV	0.207	29
chr18	78046119	T	A	splicing	Siglec15	.	0.2	30
chr2	25681381	A	C	intronic	Lcn6	.	0.194	31
chr3	107495693	C	A	exonic	Slc6a17	synonymous_SNV	0.194	31
chr7	100372422	A	T	UTR5	C2cd3	.	0.188	32
chr10	75508955	T	A	exonic	Gucd1	nonsynonymous_SNV	0.188	32
chr11	78751636	G	A	exonic	Ccnq	synonymous_SNV	0.182	11
chr3	49756214	G	A	exonic	Pcdh18	synonymous_SNV	0.143	84
chr17	47880270	G	T	exonic	Foxp4	nonsynonymous_SNV	0.667	6
chr16	17911303	C	A	exonic	Ess2	nonsynonymous_SNV	0.571	7
chr3	96582056	G	T	intronic	Polr3gl	.	0.5	8
chr9	78587892	C	A	exonic	Slc17a5	nonsynonymous_SNV	0.5	8
chr17	34000065	T	C	intronic	H2-K1	.	0.455	22
chr17	34000067	A	G	intronic	H2-K1	.	0.455	22
chr13	17695703	G	A	exonic	Mplkip	synonymous_SNV	0.444	9
chr12	86882309	G	T	exonic	Irf2bpl	nonsynonymous_SNV	0.429	7
chr5	28071573	C	A	exonic	Insig1	synonymous_SNV	0.4	5
chr8	95056803	C	A	intronic	Drc7	.	0.4	5
chr9	120120732	G	T	exonic	Slc25a38	nonsynonymous_SNV	0.4	10
chr9	120120757	C	T	exonic	Slc25a38	synonymous_SNV	0.4	10
chr17	34000075	G	T	intronic	H2-K1	.	0.4	20
chr17	35686516	G	T	exonic	Ddr1	nonsynonymous_SNV	0.4	10
chr2	3512596	G	T	intronic	Hspa14	.	0.375	8
chr17	34000077	A	T	intronic	H2-K1	.	0.368	19
chr2	60495352	C	A	exonic	Pla2r1	nonsynonymous_SNV	0.353	17
chr16	5196060	T	A	splicing	Nagpa	.	0.353	17
chr7	25786027	A	T	intronic	Axl	.	0.333	9
chr8	70392079	G	A	exonic	Crtc1	stopgain	0.333	6
chr8	92360416	G	A	exonic	Irx5	synonymous_SNV	0.333	6
chr9	65135327	G	A	exonic	Igdcc4	synonymous_SNV	0.333	12
chr9	77754845	G	T	exonic	Gclc	nonsynonymous_SNV	0.333	12
chr9	78587931	C	A	exonic	Slc17a5	nonsynonymous_SNV	0.333	6
chr12	91401054	C	G	UTR5	Tshr	.	0.333	6
chr13	48490521	G	A	exonic	Zfp169	nonsynonymous_SNV	0.333	6
chr5	124124827	G	T	exonic	Pitpnm2	nonsynonymous_SNV	0.308	13
chr5	139185660	C	A	UTR3	Dnaaf5	.	0.308	13
chr5	142886699	G	T	exonic	Fbxl18	synonymous_SNV	0.308	13
chr17	37197312	C	A	exonic	Olfr94	nonsynonymous_SNV	0.308	13
chr7	141455451	G	A	exonic	Pnpla2	synonymous_SNV	0.286	7
chr8	86746321	G	A	ncRNA_exonic	Gm10638	.	0.286	14
chr9	107947178	G	T	intronic	Camkv	.	0.286	14
chr14	55063816	G	A	exonic	Zfhx2	nonsynonymous_SNV	0.286	14
chrX	7674912	C	A	exonic	Magix	nonsynonymous_SNV	0.286	14
chrX	20060459	A	T	exonic	Chst7	nonsynonymous_SNV	0.286	7
chr17	46306138	C	G	intronic	Abcc10	.	0.267	15
chr1	171282613	T	C	splicing	Usp21	.	0.257	35
chr5	125027040	G	T	exonic	Ncor2	synonymous_SNV	0.25	12
chr9	46204396	C	T	intronic	Sik3	.	0.25	8
chr16	10790932	G	T	upstream;down	Prm3;Prm2	.	0.25	8
chr19	7014100	T	A	exonic	Fermt3	nonsynonymous_SNV	0.25	8
chrX	20059911	C	T	exonic	Chst7	stopgain	0.25	8
chr2	30091675	G	A	exonic	Zdhhc12	synonymous_SNV	0.231	13
chr8	71536424	T	C	exonic	Bst2	nonsynonymous_SNV	0.231	26
chr2	26206360	C	T	exonic	Lhx3	nonsynonymous_SNV	0.222	9
chr8	119748995	C	A	intronic	Atp2c2	.	0.222	9
chr16	95990842	C	T	exonic	Psmg1	nonsynonymous_SNV	0.222	9
chr5	28166832	A	T	exonic	En2	nonsynonymous_SNV	0.214	14
chr9	63140306	C	A	intronic	Skor1	.	0.214	14
chr15	102319770	A	T	exonic	Espl1	nonsynonymous_SNV	0.211	38
chr2	28533743	C	A	splicing	Ralgds	.	0.2	10
chr7	28359981	G	T	exonic	Plekhg2	nonsynonymous_SNV	0.2	15
chr8	95328716	G	T	exonic	Zfp319	nonsynonymous_SNV	0.2	10
chr8	124525813	C	A	intronic	Cog2	.	0.2	10
chr9	46204388	C	A	intronic	Sik3	.	0.2	10
chr9	58859752	C	A	exonic	Hcn4	nonsynonymous_SNV	0.2	10
chr11	116444961	G	A	exonic	Qrich2	synonymous_SNV	0.2	10
chr14	55628848	A	G	exonic	Ipo4	synonymous_SNV	0.2	10
chr1	132528619	G	T	exonic	Cntn2	nonsynonymous_SNV	0.188	16
chr1	132316311	G	T	exonic	Nuak2	nonsynonymous_SNV	0.182	11
chr10	61207468	C	T	splicing	Adamts14	.	0.182	11
chr12	100931934	G	A	intronic	Ccdc88c	.	0.182	11
chr12	102558514	A	T	exonic	Chga	nonsynonymous_SNV	0.182	22
chr19	41206519	C	G	exonic	Tll2	nonsynonymous_SNV	0.179	28
chr8	83668812	T	A	exonic	Ptger1	nonsynonymous_SNV	0.143	21
chr17	31850977	C	A	exonic	Sik1	nonsynonymous_SNV	0.136	22

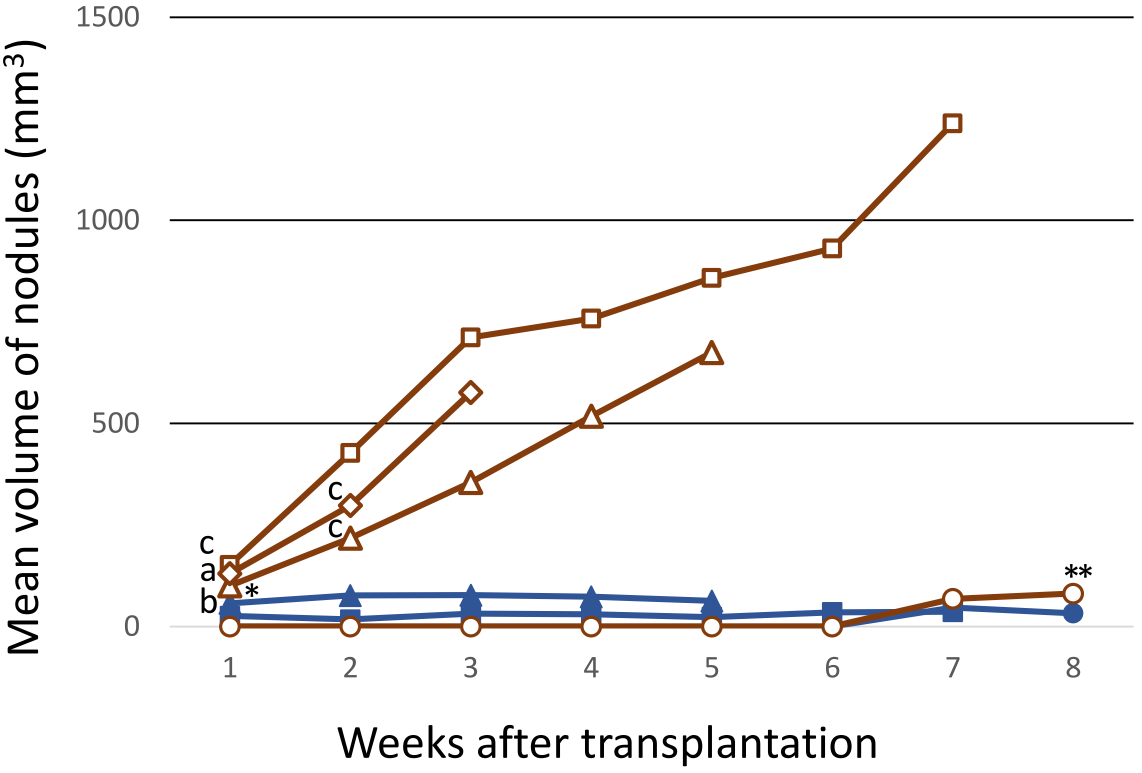

Supplementary Figure S3

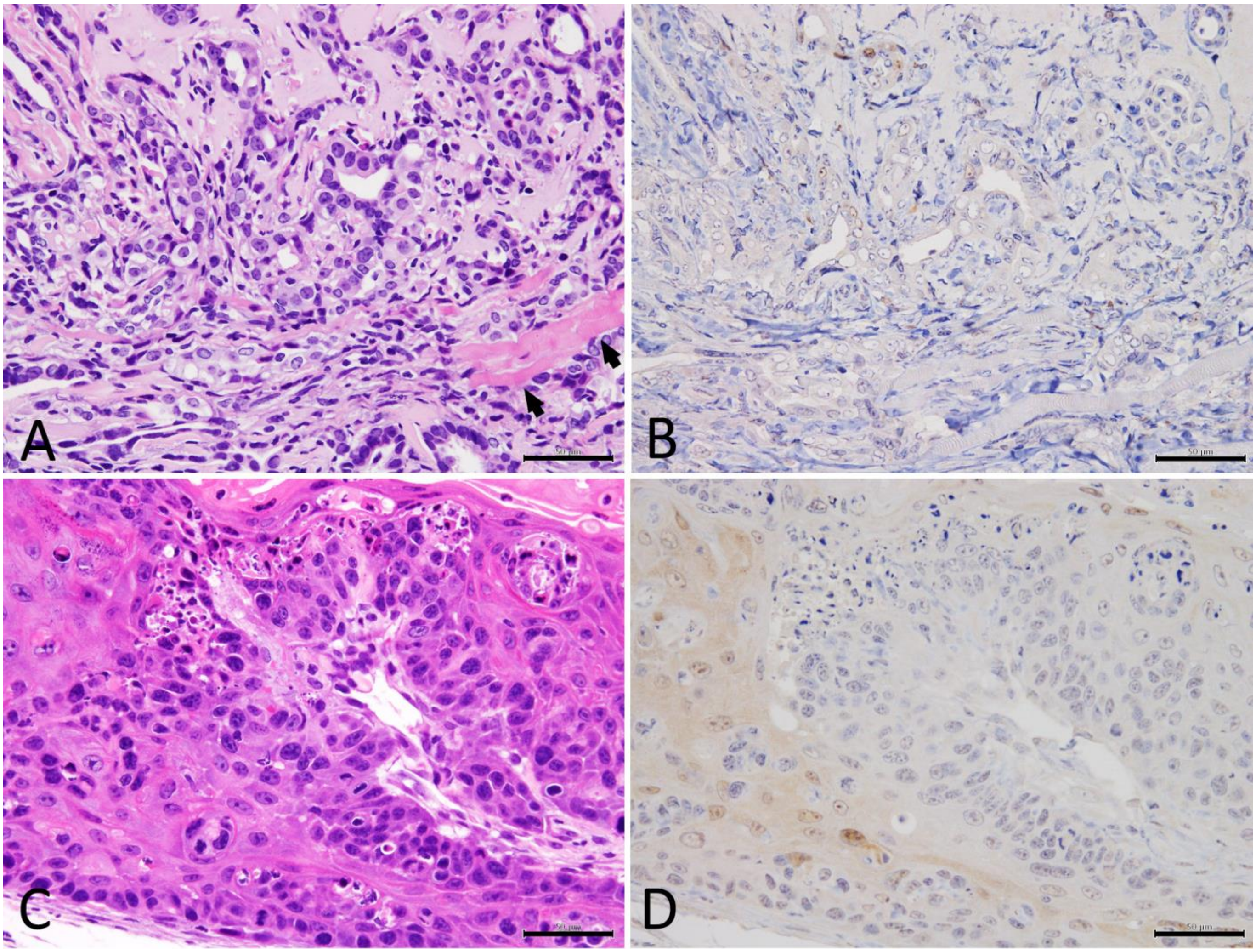

Supplementary Figure S4

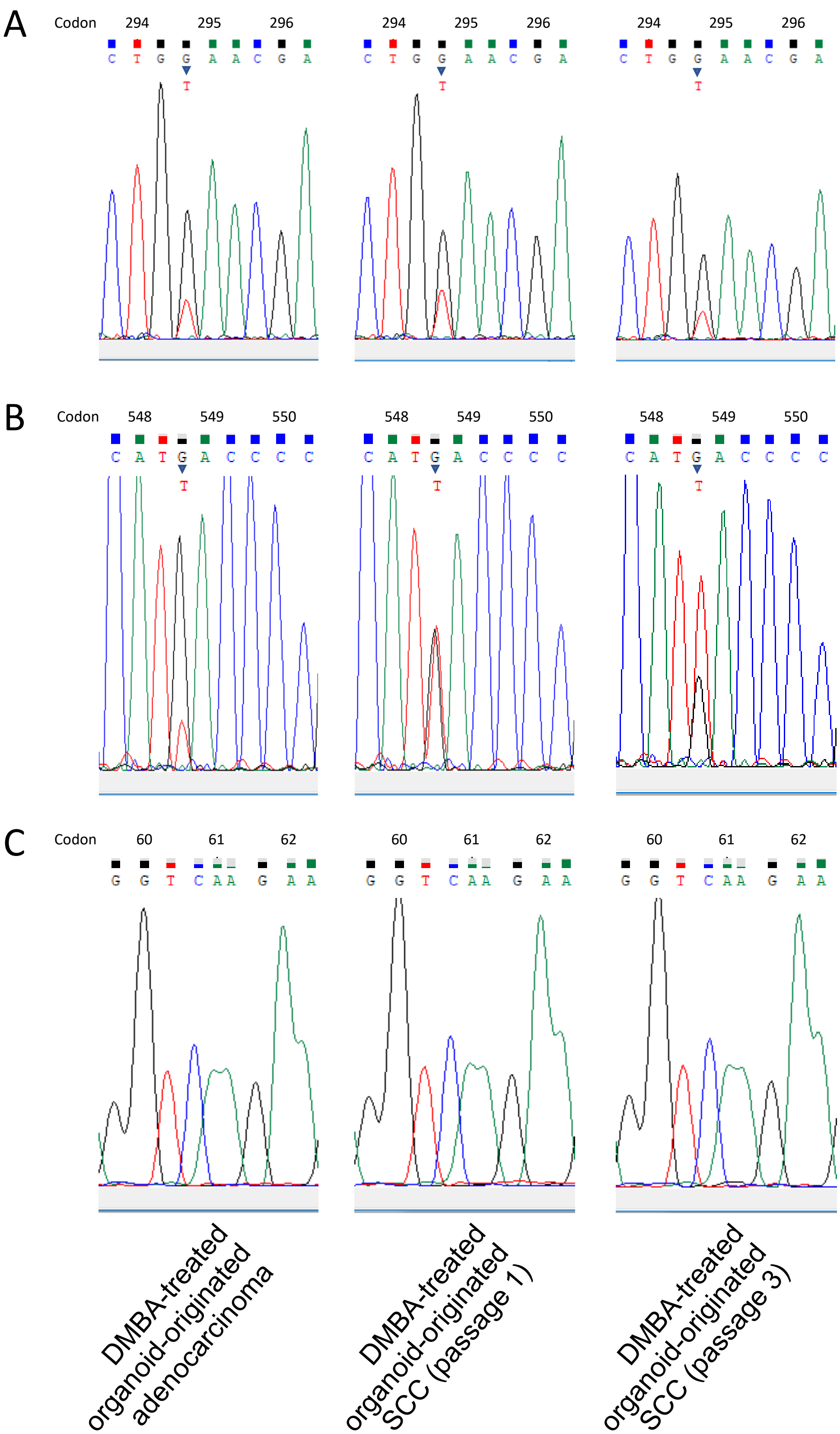

Supplementary Figure S5

A

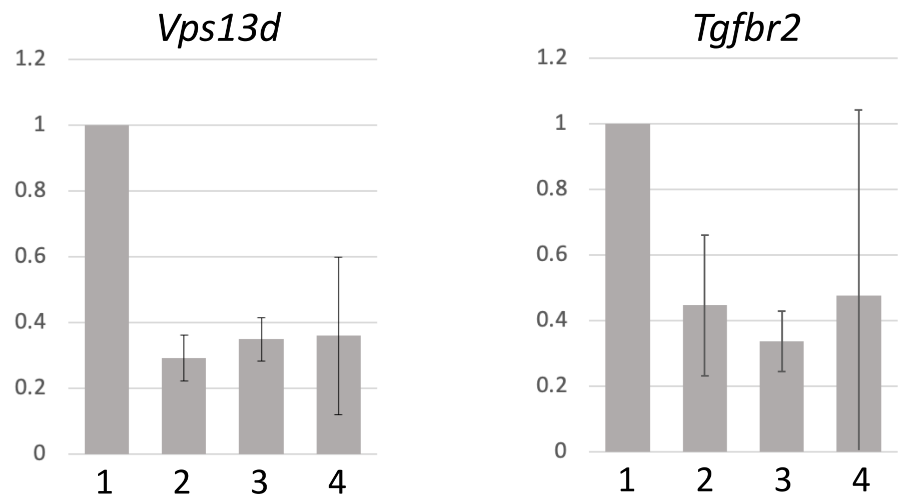

1, Normal mammary tissues from *in vivo* DMBA-treated mice (N=2)
2, Mammary adenocarcinomas induced in *in vivo* DMBA-treated mice (N=3)
3, DMBA-treated organoid-derived adenocarcinomas at passage 0 (N=3)
4, DMBA-treated organoid-derived squamous cell carcinomas at passages 3(N=3)

B

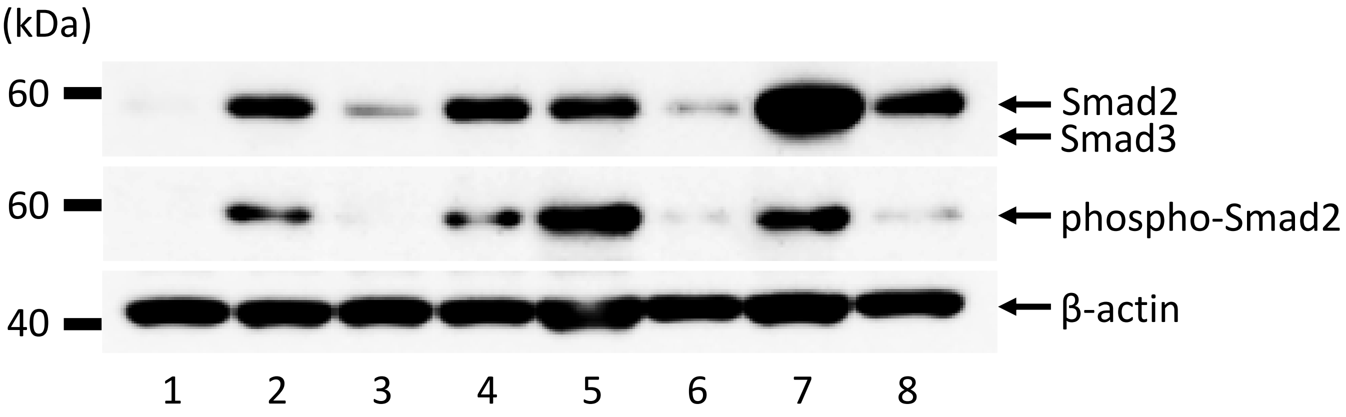

Lane 1-4 Samples from an *in vivo* DMBA-treated experiment
1, Normal mammary tissue of mouse #1
2, Mammary adenocarcinoma induced in mouse #1
3, Normal mammary tissue of mouse #2
4, Mammary adenocarcinoma induced in mouse #2
Lane 5-8 Samples from DMBA-treated organoid-derived tumors
5, Adenocarcinoma #3 at passage 0
6, Adenocarcinoma #4 at passage 0
7, Squamous cell carcinoma #5 at passage 3
8, Squamous cell carcinoma #6 at passage 3

Supplementary Figure S6
